# Sensitive quantitative analysis of the bitter glycoside amarogentin by specific monoclonal antibody-based indirect competitive enzyme-linked immunosorbent assay†

**DOI:** 10.1039/c8ra02922a

**Published:** 2018-05-14

**Authors:** Seiichi Sakamoto, Shinji Wada, Hiroyuki Tanaka, Satoshi Morimoto

**Affiliations:** Department of Pharmacognosy, Graduate School of Pharmaceutical Sciences, Kyushu University 3-1-1 Maidashi, Higashi-ku Fukuoka 812-8582 Japan s.sakamoto@phar.kyushu-u.ac.jp htanaka@phar.kyushu-u.ac.jp +81 92 642 6581 +81 92 642 6581

## Abstract

Amarogentin (AG) is a naturally occurring secoiridoid glycoside produced mainly in the plant genera *Swertia* and *Gentiana*. Extracts of these plants have a long history of use in Japan as bitter stomachics because of their strong bitterness. Because the AG content directly reflects the potential activity of the extract, the quality control of these plant extracts through the quantitative analysis of AG is important. In the present study, we aimed to develop a quantitative analysis of AG using a monoclonal antibody (MAb) against AG (MAb 1E9) in the plant family Gentianaceae. Surprisingly, production of MAb 1E9 was successfully achieved by immunizing AG–bovine serum albumin (BSA) conjugates into mice although the number of AG bound to BSA was only one. The characterization of MAb 1E9 revealed that it shows high specificity to AG, enabling the development of an icELISA for the specific determination of AG. In addition, the detectable concentration of AG in the developed icELISA ranged from 1.95 to 62.5 ng mL^−1^ with a limit of detection of 1.28 ng mL^−1^, which is approximately 30–625 times higher in sensitivity compared with the conventional HPLC method. Validation analysis revealed that the icELISA using MAb 1E9 is precise (intra-assay variation <3.9%, inter-assay variation <8.8%) and accurate (recovery rates of spiked samples were between 91.0% and 106.4%). This method can be used for the quality control of plant extracts using AG as an index.

## Abstract 2

MAb 1E9 was generated from AG–BSA conjugates possessing one AG molecule per BSA for icELISA.

## Graphical abstract

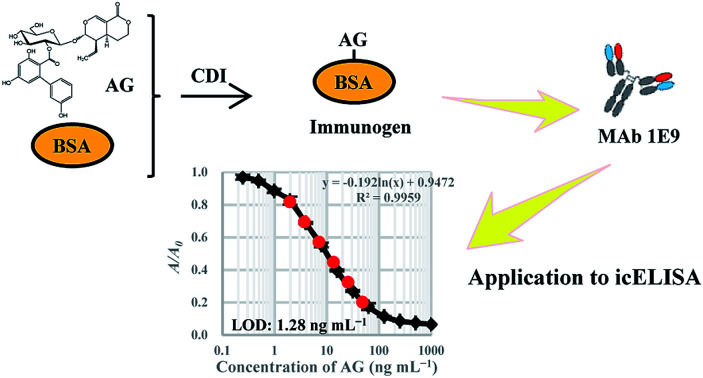

## Introduction

**Fig. 1:**
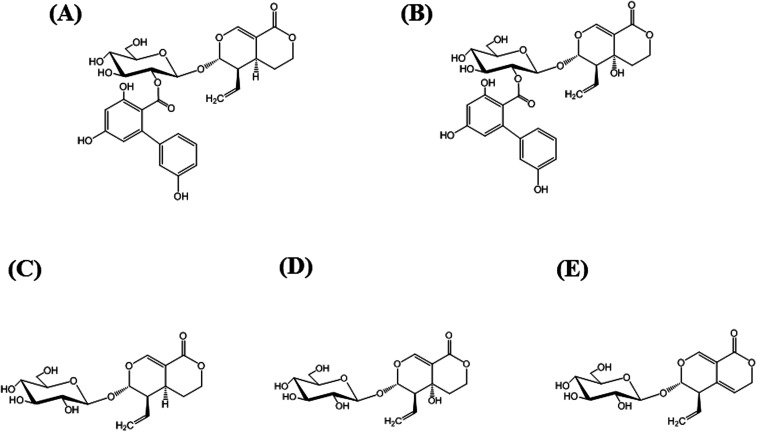
Structures of major secoiridoid glycosides, amarogentin (AG; (A)), amaroswerin (AS; (B)), sweroside (C), swertiamarin (D), and gentiopicroside (E).

Amarogentin [AG; Fig. 1(A)] is a naturally occurring secoiridoid glycoside produced mainly in plants belonging to the genera *Swertia* and *Gentiana*, both belonging to the family Gentianaceae. The extracts of members of this family have long been used as bitter stomachics because they contain bitter secoiridoid glycosides, including AG, amaroswerin (AS), swertiamarin, sweroside, and gentiopicroside (Fig. 1), all of which enhance gastrointestinal motility and the secretion of digestive juices.^1–3^ Among them, AG is known as the bitterest natural compound^4^ and the root extract of *Gentiana lutea* is registered as a bitter food additive in the List of Existing Food Additives in Japan.^5^ In addition to the activity of AG as a bitter stomachic, AG has been shown to possess various pharmacological activities, including anti-diabetic activity,^6^ anti-leishmania activity,^7,8^ and hepatoprotective activities.^9^ More recently, AG was found to induce the apoptosis of liver cancer cells by the upregulation of p53 and downregulation of human telomerase reverse transcriptase.^10^ Thus, because the AG content directly reflects the potential activity of the extract, the quality control of Gentianaceae through the quantitative analysis of AG has recently attracted much attention. Currently, quantitative analysis for AG has been reported using liquid chromatography-mass spectrometry (LC-MS),^11^ high-performance liquid chromatography (HPLC),^11,12^ ultra-performance LC-electrospray ionization/MS,^13^ and capillary electrophoresis.^14^ However, these methods require large amounts of eco-unfriendly organic solvents, costly instruments, and time-consuming sample pretreatment.

In our previous studies, we focused on the quantitative analysis of plant secondary metabolites and developed various immunoassays, including enzyme-linked immunosorbent assays (ELISAs), fluorescence-linked immunosorbent assays, and immunochromatographic strip assays. These immunoassays exhibited several advantages over traditional analytical methods, such as HPLC, in terms of simplicity, selectivity, and sensitivity because the principle is based on a specific antigen–antibody reaction.^15^

In the present study, we produced a specific monoclonal antibody (MAb) against AG (MAb 1E9) to develop an indirect competitive ELISA (icELISA) for the determination of AG. AG–bovine serum albumin (BSA) conjugates were prepared *via N*,*N*′-carbonyldiimidazole (CDI) and were administered to BALB/c mice to induce an immunoresponse. Hybridomas secreting MAb 1E9 were obtained *via* the polyethylene glycol (PEG) method, and the MAb 1E9 were purified using affinity column chromatography. It is note that MAb 1E9 was successfully produced using AG–BSA conjugates possessing only one AG molecule per BSA molecule. The systematic characterization of MAb 1E9 revealed that it possesses high specificity to AG and high sensitivity, with a limit of detection (LOD) of 1.28 ng mL^−1^. To the best of our knowledge, this is now the most sensitive analytical method for determining the AG content in plant samples. Various validation analyses, including intra- and inter-assay tests, and spiked recovery tests alongside correlation analysis using HPLC support the accuracy and precision of the developed icELISA for the quality control of Gentianaceae through the quantitative analysis of AG.

This paper demonstrated the production of MAb 1E9 and its application to icELISA.

## Experimental section

### Chemicals and reagents

Amarogentin (AG, ≥95%) was obtained from EXTRASYNTHESE (Lyon, France). Bovine serum albumin (BSA, ≥97%), human serum albumin (HSA, ≥99%), and ovalbumin (OVA, ≥98%) were purchased from Sigma-Aldrich (St. Louis, MO, USA). Freund's complete and incomplete adjuvants were obtained from Difco (Detroit, MI, USA). Goat F(ab) Anti-Mouse IgG H&L (HRP) (ab6823) was used as a secondary antibody and was purchased from Abcam (Cambridge, MA, USA). All the other chemicals were standard commercial products of analytical to reagent grade.

### Isolation of AS from *Swertia japonica*

To evaluate the specificity of MAb 1E9 to AG, structurally similar compounds were required. Because AS [Fig. 1(B)] was commercially unavailable, it was isolated from *S. japonica* (powdered; Uchida Wakanyaku Co. Ltd., Tokyo, Japan). *S. japonica* (∼200 g) were soaked in methanol (500 mL), sonicated for 1 h, and filtered. This extraction step was repeated five times, and the filtrates (∼2.5 L) were then concentrated under vacuum to obtain ∼53.5 g of extracts. The extracts were dissolved in 50% (v/v) MeOH and partitioned with hexane. The aqueous layer was then partitioned with CHCl_3_, followed by ethyl acetate, to obtain an ethyl acetate fraction (∼4.3 g) containing AS. Reverse-phase chromatography using Cosmosil 75C_18_-OPN (Nacalai Tesque, Kyoto, Japan) with 50% (v/v) MeOH and silica gel 60 (0.063–0.200 mm; Merck, Darmstadt, Germany) chromatography with a mixture of CHCl_3_ and MeOH (CHCl_3_/MeOH = 8/2) was performed to obtain AS (∼210 mg). NMR spectra were recorded on a Varian Unity 500 plus apparatus (Varian, Palo Alto, CA, USA) operating at 500 MHz for ^1^H NMR and 125 MHz for ^13^C NMR in methanol-*d*_4_.

### Animals

BALB/c mice (5 weeks old, male) were purchased from KBT Oriental Co., Ltd. (Saga, Japan). A standard diet (MF; Oriental Yeast Co., Tokyo, Japan) and water were provided *ad libitum*. The animal experiment conducted in the present study was approved by the Committee on the Ethics of Animal Experiments, Kyushu University (approval no.: A30-003-0), and the experimental procedures were performed in accordance with the Guidelines for Animal Experiments of the Graduate School of Pharmaceutical Sciences, Kyushu University.

### Preparation of plant samples

Seven kinds of Gentianaceae were analyzed using icELISA and HPLC. *S. japonica* (powdered), *S. japonica* (whole plant), *G. lutea* (powdered), and *G. scabra* (powdered) were commercially obtained from Uchida Wakanyaku Co. Ltd., (Tokyo, Japan). *S. japonica* (whole plant, Nagano, Japan), *G. scabra* I (root), and *G. scabra* II (root; Ibaraki, Japan) were obtained from voucher specimens deposited at the Herbarium of the Faculty of Pharmaceutical Sciences, Kyushu University. Powdered samples were measured without any treatment, whereas whole plants and roots were ground into a fine powder and sieved through a 300 μm mesh filter. Plant powder (50 mg) was measured and immersed in MeOH (1 mL). Extraction was performed by sonication for 20 min, and the supernatant, after centrifugation at 10 000 rpm for 10 min, was collected in small test tubes. This step was repeated three times. The combined MeOH extract (3 mL) was concentrated to dryness under flowing nitrogen. The residue was then dissolved in MeOH (1.0 mL) and used as a sample for both icELISA and HPLC.

### Preparation of AG–BSA, AG–HSA, and AG–OVA conjugates

AG possesses a hydroxyl group; therefore, AG was conjugated to proteins (BSA, HSA, and OVA) *via* CDI, which is a zero-length cross-linking reagent.^16^ For the AG–BSA conjugates used as immunogens, AG (3.1 mg) and CDI (3.0 mg) were dissolved in dimethylformamide (0.6 mL), which was stirred at 30 °C for 3 h. Subsequently, this solution was added dropwise to another solution, where BSA (4.2 mg) was dissolved in distilled water (2.7 mL). The resulting mixture was then stirred at 30 °C for 20 h. The reaction mixture was dialyzed five times against distilled water at 4 °C for 2 days and then lyophilized to obtain AG–BSA conjugates (3.9 mg). AG–HSA (3.8 mg) and AG–OVA (3.6 mg) conjugates were prepared in the same manner using HSA (3.9 mg) and OVA (4.1 mg), respectively. All conjugates were dissolved in 50 mM Tris–HCl (pH 8.0) containing 8 M urea at 20 mg mL^−1^ and stored at −20 °C until use.

### Production of MAb 1E9

Immunization of BALB/c mice (5 weeks old, male) was performed with AG–BSA conjugates every 2 weeks, as described previously.^17^ At the first and second immunizations, Freund's complete and incomplete adjuvants were used to enhance the immune response by forming an emulsion with the immunogen (AG–BSA conjugates). The resulting emulsions, corresponding to 50 μg of AG–BSA conjugates, were administered intraperitoneally. Booster shots were administered three times by immunization with AG–BSA conjugates (100 μg) without using adjuvants. On the fifth day after the final booster, the splenocytes were prepared and fused with SP2/0 mice myeloma cells using the PEG method. The fused cells were then cultured in a selection medium containing enriched RPMI 1640-Dulbecco's-Ham's F12 (eRDF; Kyokuto Pharmaceutical Industrial Co., Tokyo, Japan) medium supplemented with hypoxanthine–aminopterin–thymidine (HAT), RD-1 additives (Kyokuto Pharmaceutical Industrial Co., Tokyo, Japan), and 10% (v/v) fetal calf serum (FCS; Gibco-Invitrogen, Carlsbad, CA, USA) at 37 °C in a humidified incubator and a 5% CO_2_ atmosphere. Hybridomas secreting anti-AG antibody were then cloned by the limited dilution method and selected by direct ELISA and icELISA. The selected hybridomas (1E9) were scaled up in the same buffer without HAT until the volume became 1 L. Finally, the medium was exchanged to a serum-free medium (without FCS) and further cultured for 1 week.

Purification of MAb 1E9 was performed using affinity column chromatography. Briefly, the culture medium containing MAb 1E9 was centrifuged, and the pH of the supernatant was adjusted to pH 7.0 with 1 M Tris–HCl buffer (pH 9.0). The culture medium was then filtered, and the filtrates were subjected to chromatography on a Protein G Sepharose 4 Fast Flow (GE Healthcare Bio-Science AB, Uppsala, Sweden) column equilibrated with 10 mM phosphate buffer (pH 7.0). The bound proteins were then washed with 10 mM phosphate buffer (pH 7.0) and eluted with 100 mM citrate buffer (pH 2.7). Ten test tube fractions containing MAb 1E9 were obtained. To avoid inactivation of the eluted MAb 1E9, the eluates (4.0 mL) were immediately neutralized with 1 M Tris–HCl buffer (pH 9.0; 1.4 mL). Subsequently, all fractions were collected, concentrated, dialyzed against distilled water at 4 °C for 1 week, and lyophilized to yield 79.0 mg of MAb 1E9.

### Indirect ELISA and icELISA using MAb 1E9

The binding reactivity of MAb 1E9 was evaluated by ELISA using AG–HSA as the coated antigen. The reactivity against AG–HSA conjugates and free AG molecules was investigated by indirect ELISA and icELISA, respectively. In both ELISA tests, AG–HSA conjugates (5 μg mL^−1^) in 50 mM carbonate buffer (pH 9.6; 100 μL per well) were immobilized to the surface of a 96-well immunoplate (Nunc, Maxisorb, Roskilde, Denmark) by incubation at 37 °C for 1 h. After washing the plate, the wells were then treated with PBS containing 5% (w/v) skimmed milk (300 μL per well) at 37 °C for 1 h to block and reduce non-specific adsorption of other proteins. The following step, using MAb 1E9, was different between indirect ELISA and icELISA. For indirect ELISA, various concentrations of MAb 1E9 (100 μL per well) in PBS containing 0.05% (v/v) Tween 20 (PBS-T) were allowed to react with the coated AG–HSA conjugates. For icELISA, free AG molecules (50 μL per well) in 5% (v/v) MeOH were mixed with MAb 1E9 (50 μL per well; 900 ng mL^−1^) in PBS-T, leading to a competitive reaction between free AG molecules and AG of coated AG–HSA conjugates against MAb 1E9. After incubation at 37 °C for 1 h and a subsequent washing step, MAb 1E9 binding to the immobilized AG–HSA conjugates was reacted with Goat F(ab) Anti-Mouse IgG H&L (HRP) (100 μL per well), which was 5000 times diluted with PBS-T, at 37 °C for 1 h. Finally, the 0.3 mg mL^−1^ 2,2′-azino-bis(3-ethylbenzothiazoline-6-sulfonic acid)diammonium salt substrate solution in 100 mM citrate buffer (pH 4.0) supplemented with 0.003% (v/v) H_2_O_2_ (100 μL per well) was added and incubated at 37 °C for 15 min to develop color.

All the washing steps were performed thrice using PBS-T between each step. The developed color was detected based on the absorbance at 405 nm using a microplate reader (Multiskan™ FC microplate photometer, Thermo Fisher Scientific, Inc., Waltham, MA, USA). The cross-reactivities (CRs) of MAb 1E9 were evaluated using the formula described by Weiler and Zenk as follows:^18^
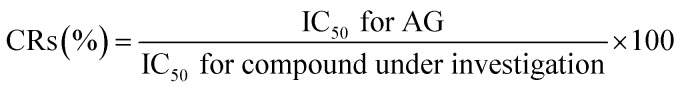


### Recovery of spiked AG

Various amounts of AG (7.5, 15, and 30 μg) were spiked into MeOH solution (1 mL) extracted from *S. japonica* (powdered). In the unspiked samples, 95.86 μg of AG was found per 50 mg of dried sample by icELISA. After spiking with AG standards, the amounts of AG were determined using the developed icELISA and the recovery rate was calculated as follows:
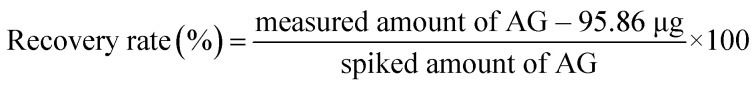


### HPLC analysis

HPLC analysis was performed according to the method by Amakura *et al.*, with slight modification.^12^ An LC-10AD VP HPLC pump connected to a SPD-10A UV/VIS detector (254 nm) and C-R8A chromatopac, all of which were Shimadzu products (Kyoto, Japan), were used in the analysis. After the samples (20 μL) were subjected to a reversed-phase column (Cosmosil-packed 5C_18_ AR II 4.6 mm × 150 mm, 5 μm particle size, Nacalai Tesque, Kyoto, Japan), equilibrated with a mobile phase comprising 20% (v/v) acetonitrile with 0.5% (v/v) acetic acid solution, the eluates were analyzed for 20 min. AG was detected at the retention time of ∼17.0 min when the flow rate was 1.0 mL min^−1^. The calibration curve for AG was drawn in the concentration range of 0.049–12.5 μg mL^−1^ with a LOD of 0.23 μg mL^−1^. All sample analyses were performed in triplicate.

## Results and discussion

### Determination of hapten numbers by MALDI-TOF-MS

The number of AG molecules bound to the BSA carrier protein was evaluated by MALDI-TOF-MS analysis (Bruker Autoflex III). In this analysis, the BSA and AG–BSA conjugates appeared at *m*/*z* 66 451 and *m*/*z* 67 224, respectively (Fig. S1†). By taking the molecular weight of AG (586.54) into account, at least one molecule of AG was found to be conjugated to a BSA molecule. Approximately 40 years ago, the appropriate hapten numbers had already been investigated by Erlanger for the production of anti-hapten antibodies using BSA conjugates and he concluded that between 8 and 25 molecules were optimal.^19^ However, in our recent reports, highly specific and highly sensitive antibodies were successfully produced against harringtonine,^20^ homoharringtonine,^21^ and monocrotaline,^22^ despite only having hapten numbers of 2 per BSA molecule. Thus, AG–BSA conjugates obtained herein were used as immunogens for further antibody production.

### Production and typing of MAb 1E9

**Fig. 2:**
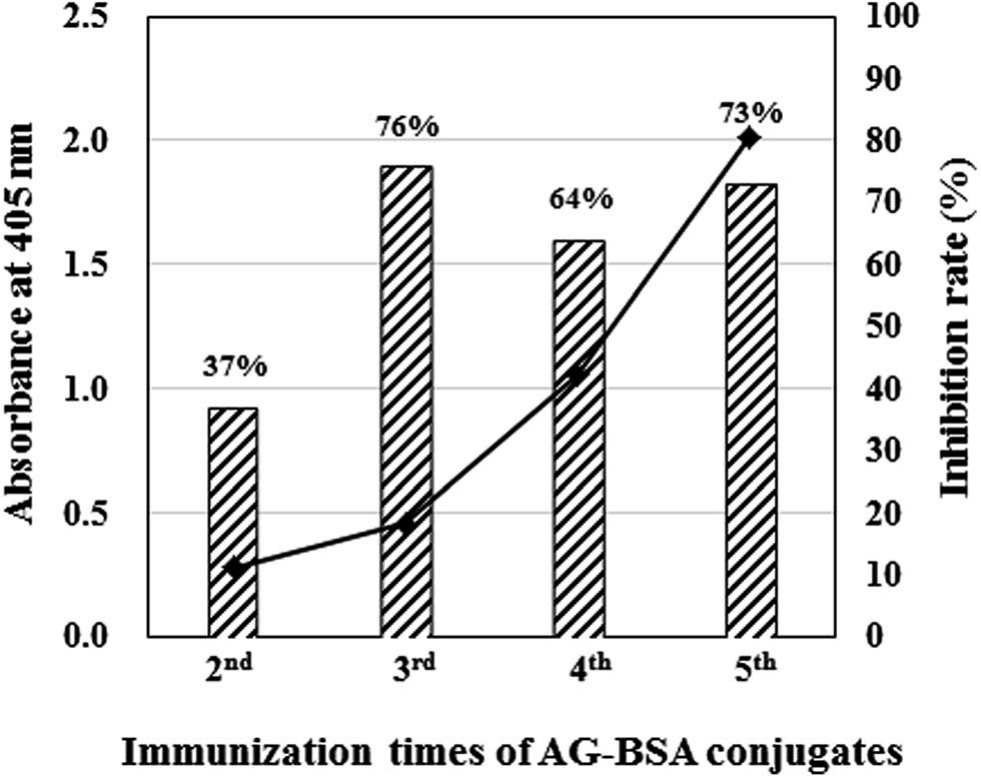
Antibody titer and its inhibition rate against AG evaluated by ELISAs. The closed squares and hatched bar graphs indicate absorbance at 405 nm evaluated by indirect ELISA and inhibition rate against AG (50 μg mL^−1^). In both ELISAs, AG–OVA conjugates (1 μg mL^−1^) were used as coated antigen, and 100 times diluted antisera with PBS-T were used as primary antibody.

On the fourth day after immunization, mouse serum was obtained from the tail vein of BALB/c mice, and the titer and inhibitory activity against AG (50 μg mL^−1^) in the serum were investigated by indirect ELISA and icELISA, respectively (Fig. 2). Interestingly, despite the hapten number of 1, the antibody titer increased as the immunization times increased. In addition, icELISA revealed that the antibody in the serum obviously inhibits free AG. Since the antibody titer (OD_405_ = 2.01) and inhibition rate against AG (73%) increased enough to be used in ELISA after the fifth immunization (Fig. 2), cell fusion between splenocytes and SP2/0 mice myeloma cells was performed using the PEG method. After several screenings, only one hybridomas (1E9) secreting anti-AG antibody was obtained and scaled up to purify MAb 1E9.

The isotype of MAb 1E9 was identified using an IsoStrip Mouse Monoclonal Antibody Isotyping Kit (Roche Diagnostics, Mannheim, Germany), and this revealed that the MAb 1E9 was an IgG1 that possessed a κ light chain.

### Optimization of icELISA using MAb 1E9

**Fig. 3:**
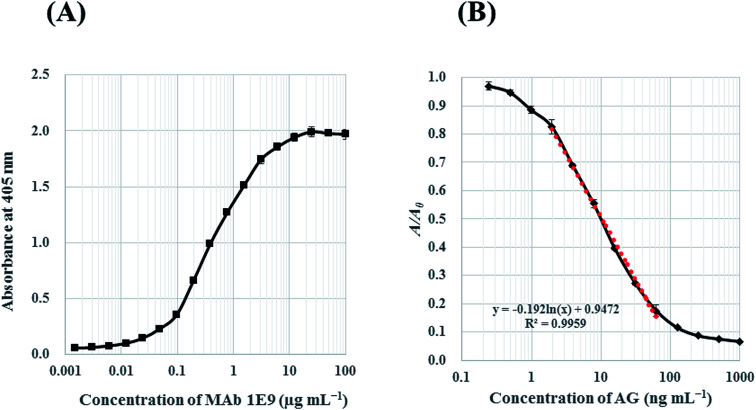
Reactivity response curves in indirect ELISA against AG–HSA conjugates (A) and icELISA against free AG (B). In both ELISAs, concentration of AG–HSA conjugates was 5 μg mL^−1^. In icELISA (B), *A*_0_ and *A* corresponds the absorbance in the absence and presence of AG. A dotted line represents linearized curve for AG with the determination range from 1.95 to 62.5 ng mL^−1^.

The binding reactivity of MAb 1E9 against AG–HSA conjugates (5 μg mL^−1^) was analyzed by indirect ELISA. The absorbance against log[MAb 1E9] was plotted for the reactivity response curve. The concentration of MAb 1E9 positively correlated with the absorbance in a logarithmic manner [Fig. 3(A)]. This result revealed that 450 ng mL^−1^ of MAb 1E9 gives an absorbance of ∼1.0 in the indirect ELISA test, suggesting that 900 ng mL^−1^ is optimal for MAb 1E9 as a primary antibody in icELISA because the volume of primary antibody in icELISA (50 μL) should be half of that in an indirect ELISA (100 μL), as mentioned above.

Then, the inhibitory activity of MAb 1E9 against free AG was analyzed by icELISA. The AG standard was serially diluted with 5% (v/v) MeOH, and applied (50 μL per well) to the immunoplate coated with AG–HSA conjugates. MAb 1E9 diluted with PBS-T (900 ng mL^−1^; 50 μL well^−1^) was then added and incubated to allow for competition between free AG/AG–HSA conjugates and MAb 1E9. The increased free AG led to a decrease in the amount of MAb 1E9 that could bind to AG–HSA conjugates, and *vice versa*; thus, the absorbance decreased as the MAb 1E9 concentration increased in a logarithmic manner. In this icELISA, the detectable concentration of AG was in the range of 1.95–62.5 ng mL^−1^ with a LOD of 1.28 ng mL^−1^ [Fig. 3(B)]. When the sensitivity of the developed icELISA was compared with that of HPLC systems on the basis of LOD, it was found that this icELISA test is ∼180 times more sensitive than our developed HPLC system (LOD = 0.24 μg mL^−1^) and ∼30–625 times more sensitive than previously reported HPLC systems (Table 1).

**Table 1:** Comparison of LOD between developed icELISA and reported HPLC system

Method	LOD
icELISA	1.28 ng mL^−1^
HPLC	37 ng mL^−1^ (ref. 11)
	0.8 μg mL^−1^ (ref. 12)

### CR test of MAb 1E9

Specificity and sensitivity are two of the most important factors in quantitative analysis. Specificity in ELISA can be evaluated from cross-reaction of antibodies. To investigate the specificity of MAb 1E9 against AG, structurally similar compounds to AG were necessary. Because AS [Fig. 1(B)] was commercially unavailable, it was isolated from powdered *S. japonica* and identified as AS by comparison with reported ^1^H and ^13^C NMR spectroscopic data.^23^ For screening, the first icELISA was performed using 41 kinds of natural products at a concentration of 50 μg mL^−1^ (Fig. S2†). This screening revealed that MAb 1E9 shows no recognition against 37 kinds of compounds that do not structurally resemble AG. In contrast, MAb 1E9 exhibited clear inhibition against four secoiridoid glycosides, which include AS, swertiamarin, sweroside, and gentiopicroside (Fig. 1); therefore, these four secoiridoid glycosides were serially diluted with 5% (v/v) MeOH and applied to a second icELISA for further CR testing. Their CRs were calculated from the ratio of the IC_50_ of AG to that of the four test compounds. CR testing revealed that MAb 1E9 possesses high specificity to AG because the CRs against the four secoiridoid glycoside test compounds were <3% (Table 2). The structural difference between AG and sweroside is the trihydroxy-biphenyl carboxylic acid moiety. Considering the drastic decrease in CR against sweroside (CRs: 0.84%), it can be stated that the trihydroxy-biphenyl carboxylic acid moiety plays an important role in conformational recognition by MAb 1E9. Moreover, the recognition by MAb 1E9 against AG (CRs: 100%) was found to be different from that against AS (CRs: 2.28%), although the structural difference between AG and AS is only the hydroxyl group at the C5 position; this suggests that this functional group contributes greatly to MAb 1E9 recognition as well as the trihydroxy-biphenyl carboxylic acid moiety. Collectively, MAb 1E9 specifically recognizes the entire AG molecule.

**Table 2:** Cross-reactivity (CR) of MAb 1E9 against secoiridoid glycosides

Class	Compound	CR (%)
Secoiridoid glycosides	Amarogentin (AG)	100.00
	Amaroswerin (AS)	2.28
	Sweroside	0.84
	Swertiamarin	0.04
	Gentiopicroside	0.04

This highly specific recognition of AG by MAb 1E9 suggests that it can be applied to the specific determination of AG using icELISA.

### Validation analysis of icELISA using MAb 1E9

The precision of the icELISA using MAb 1E9 was investigated using intra- and inter-assay tests, while its accuracy was examined *via* a recovery test.

The intra-assay precision was evaluated by observing the 405 nm absorbance variation from well to well in the same plate (*n* = 6). The inter-assay precision was evaluated by observing the variations from different plates (*n* = 5) at six AG concentrations within the detectable range (62.5, 31.3, 15.6, 7.81, 3.91, and 1.95 ng mL^−1^). The maximum coefficient of variation in the intra-assay test was 3.86% whereas that in the inter-assay test was 8.75%, indicating that the developed icELISA possesses high repeatability for the determination of AG (Table 3).

**Table 3:** Intra- and inter-assay precision tests of icELISA using MAb 1E9^a^

Concentration of AG (ng mL^−1^)	CV (%)	
	Intra-assay (*n* = 6)	Inter-assay (*n* = 5)
62.5	3.86	8.75
31.3	3.07	6.48
15.6	1.28	4.39
7.81	1.87	3.60
3.91	1.59	3.82
1.95	1.50	2.88

a All values represent coefficient of variation (CV) calculated from formula as follow: CV = standard deviation (S.D.)/mean × 100.

The accuracy of icELISA was further analyzed *via* the recovery test. In the recovery test, various amounts of AG (7.5, 15, and 30 μg) were spiked into MeOH extracts obtained from powdered *S. japonica* and the recovery rate was calculated using the measured amount of AG by icELISA and the spiked amount (Table 4). These results show that the most spiked AG sample is recovered within rates between 91.0% and 106.4%, supporting the accuracy of the developed icELISA and the usefulness of MAb 1E9 as a tool for reliable AG determination in plant samples.

**Table 4:** Recovery of spiked AG from MeOH extract of *S. japonica* determined by icELISA

Spiked amount of AG (μg)	Measured amount of AG^a^ (μg per 50 mg dry wt)	CV^b^ (%)	Expected amount of AG (μg per 50 mg dry wt)	Recovery^c^ (%)
0	95.86 ± 8.56	8.93		
7.5	102.68 ± 1.36	1.32	103.36	91.0
15	111.82 ± 8.83	7.90	110.86	106.4
30	127.61 ± 1.51	1.19	125.86	105.8

a All values were mean ± standard deviation (S.D.) from triplicate samples for each level.

b Coefficient of variation (CV) = S.D./mean × 100.

c Recovery (%) = (measured amount – 95.86 μg per spiked mount) × 100.

### Determination of AG in members of the plant family Gentianaceae by icELISA and HPLC

The AG content in members of the plant family Gentianaceae (*S. japonica*, *G. lutea*, and *G. scabra*) was determined using the newly developed icELISA method and HPLC (Table 5). The AG content in *S. japonica*, as determined by icELISA, was positively correlated with that determined by HPLC. Regarding *G. lutea*, AG was not determined by HPLC because the AG content was below LOD (0.24 μg mL^−1^); however, the high sensitivity of icELISA enabled AG detection. With respect to *G. scabra*, small amounts of AG were detected by icELISA but no AG was detected by HPLC. To date, there have been no reports regarding the presence of AG in *G. scabra*; however, *G. scabra* has been reported to contain much gentiopicroside and sweroside (1–3% wt/dry wt).^24^ Note that gentiopicroside and sweroside had slight CRs to MAb 1E9 (Table 2). These slight CRs, in contrast to the significant amounts of gentiopicroside and sweroside in *G. scabra* may result in a false positive for the detection of AG by icELISA. However, the presence of AG in *G. scabra* needs to be investigated further.

**Table 5:** Determination of AG in the members of plant family Gentianaceae by developed icELISA and HPLC^a^

Sample name	icELISA		HPLC	
	% (wt/dry wt)	CV (%)	% (wt/dry wt)	CV (%)
*S. japonica* (powdered)	1.26 × 10^−1^ ± 6.96 × 10^−3^	5.51	0.886 × 10^−1^ ± 2.26 × 10^−3^	2.55
*S. japonica* (whole plant)	1.95 × 10^−1^ ± 7.63 × 10^−3^	3.91	1.33 × 10^−1^ ± 3.58 × 10^−3^	2.70
*S. japonica* (whole plant, Nagano, Japan)	1.17 × 10^−1^ ± 7.11 × 10^−3^	6.07	0.811 × 10^−1^ ± 0.963 × 10^−3^	1.19
*G. lutea* (powdered)	3.91 × 10^−2^ ± 2.38 × 10^−3^	6.10	Not quantitated	
*G. scabra* (powdered)	2.61 × 10^−3^ ± 5.08 × 10^−5^	1.94	Not detected (ND)	
*G. scabra* I (root)	9.45 × 10^−4^ ± 7.53 × 10^−5^	7.97	ND	
*G. scabra* II (root, Ibaraki, Japan)	3.80 × 10^−3^ ± 1.19 × 10^−4^	3.13	ND	

a CV represents coefficient of variation. All values were mean ± standard deviation (S.D.) from triplicate samples.

## Conclusions

In the present study, MAb 1E9, which is highly specific to AG, was successfully prepared using AG–BSA conjugates despite only one AG being bound to BSA. Considering our recent reports about specific MAbs prepared using BSA conjugates containing only two target molecules,^20–22^ very low hapten numbers may be sufficient for producing highly specific antibodies although some study concluded between 8 and 25 hapten number were optimal.^19,25^ A sensitive icELISA using MAb 1E9 was developed with a LOD of 1.28 ng mL^−1^, enabling the determination of small amounts of AG in members of the plant family Gentianaceae without the need for complicated pretreatment, which is commonly required in other analytical methods. MAb 1E9, which is specific to AG, could be useful for the quality control of Gentianaceae through quantitative AG analysis using the newly developed icELISA.

## Conflicts of interest

The authors declare no competing financial interest.

## Supplementary Material

RA-008-C8RA02922A-s001
