# Supplementary material for: Sensitive quantitative analysis of the bitter glycoside amarogentin by specific monoclonal antibody-based indirect competitive enzyme-linked immunosorbent assay
Source: RSC Adv. 2018 May 14;8(31):17410–6. doi: 10.1039/c8ra02922a (PMC9080458; doi:10.1039/c8ra02922a)
Supplement: RA-008-C8RA02922A-s001 [file RA-008-C8RA02922A-s001.pdf]

Electronic Supplementary Material (ESI) for RSC Advances.
This journal is © The Royal Society of Chemistry 2018

Full paper

Sensitive quantitative analysis of the bitter glycoside, amarogentin by specific monoclonal antibody-based indirect competitive enzyme-linked immunosorbent assay

Seiichi Sakamoto,[a,*] Shinji Wada,[a] Hiroyuki Tanaka,[a,*] Satoshi Morimoto [a]

[a] *Graduate School of Pharmaceutical Sciences, Kyushu University, 3-1-1 Maidashi, Higashi-ku, Fukuoka 812-8582, Japan*

Running title: Sensitive detection of amarogentin by icELISA

*Corresponding author:

Seiichi Sakamoto and Hiroyuki Tanaka

Department of Pharmacognosy, Graduate School of Pharmaceutical Sciences,

Kyushu University; 3-1-1 Maidashi, Higashi-ku, Fukuoka 812-8582, Japan

E-mail: s.sakamoto@phar.kyushu-u.ac.jp (Sakamoto S) htanaka@phar.kyushu-u.ac.jp (Tanaka H)

Tel. & Fax: +81 92 642 6581

Supplementary information

Determination of hapten number of AG-BSA conjugates by matrix-assisted laser desorption/ionization time-of-flight mass spectrometry (MALDI-TOF-MS)

The hapten numbers of the AG-BSA conjugates were evaluated by MALDI-TOF-MS in accordance with our previous method.[1] Primarily, AG-BSA conjugates were serially diluted with distilled water at a final concentration of 20, 10, 5, and 2.5 mg mL^{-1}. They were then mixed with matrix solution of sinapinic acid saturated in a mixture solution of 0.15% (v/v) trifluoroacetic acid and acetonitrile in a ratio of 2 to 1. The resulting mixtures (2 μL) were applied onto an MTP 384 ground steel target plate after centrifugation (Bruker Daltonics, Bremen, Germany) and the samples were subjected to MALDI-TOF-MS (BRUKER Autoflex III, Bruker Daltonics, Bremen, Germany). Nitrogen laser (337 nm, 200 Hz maximum firing rate) was used for irradiation and the spectrum was directly recorded in linear positive high-mass mode with a mass range of 10,000 to 200,000 Da. The data were then analyzed using flexControl software (Bruker Daltonics, Bremen, Germany).

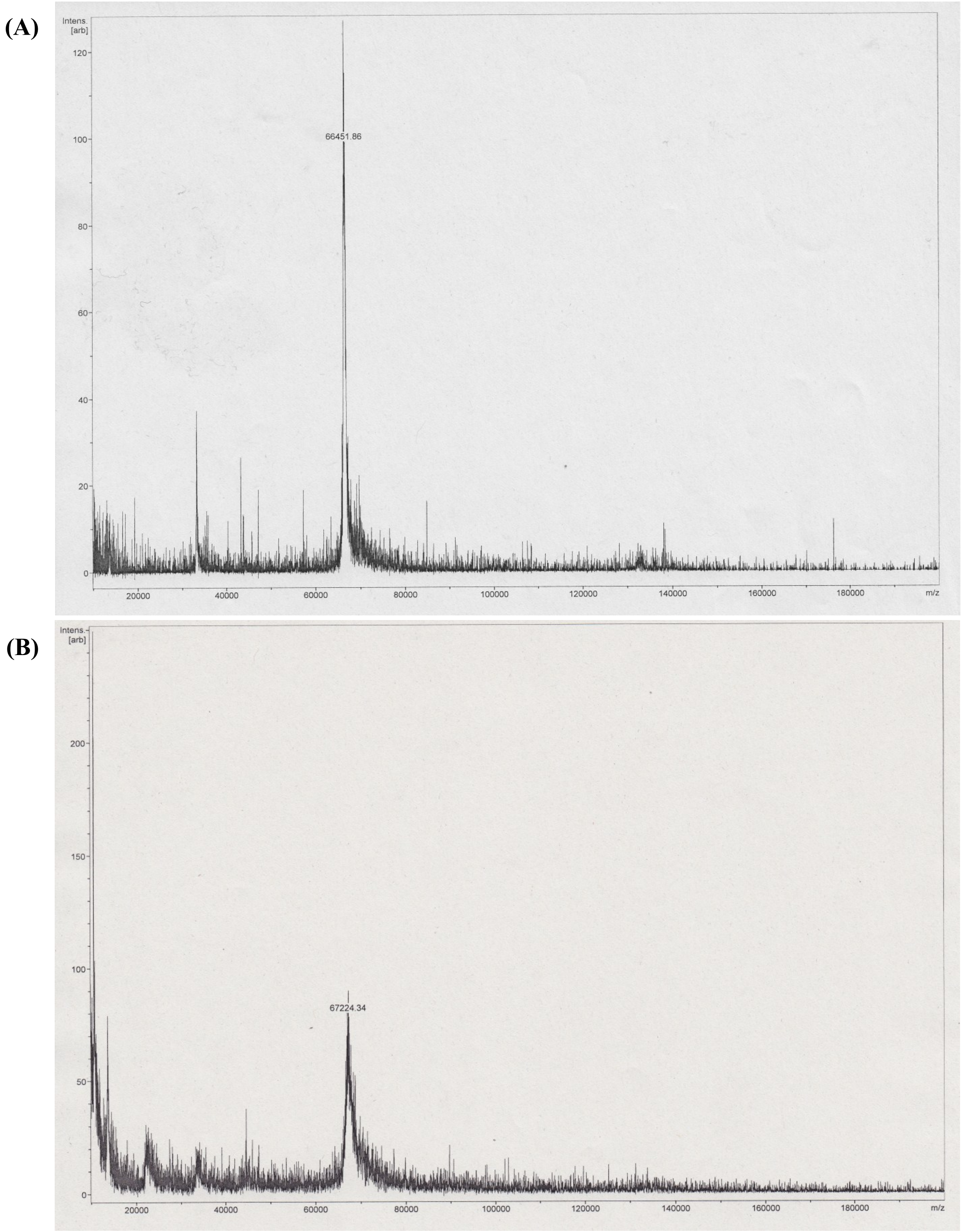

Figure S1 MALDI-TOF-MS spectrum of BSA (A) and AG-BSA conjugates (B) for determination of hapten numbers.

Screening of test compounds for further CRs test

To investigate CRs of MAb 1E9, screening was conducted by icELISA using 41 kinds of natural products. The samples were prepared in 5% (v/v) MeOH at the final concentration of 50 μg mL^{-1} and the compounds showing the inhibition rate above 50% were further analyzed in the CRs test.

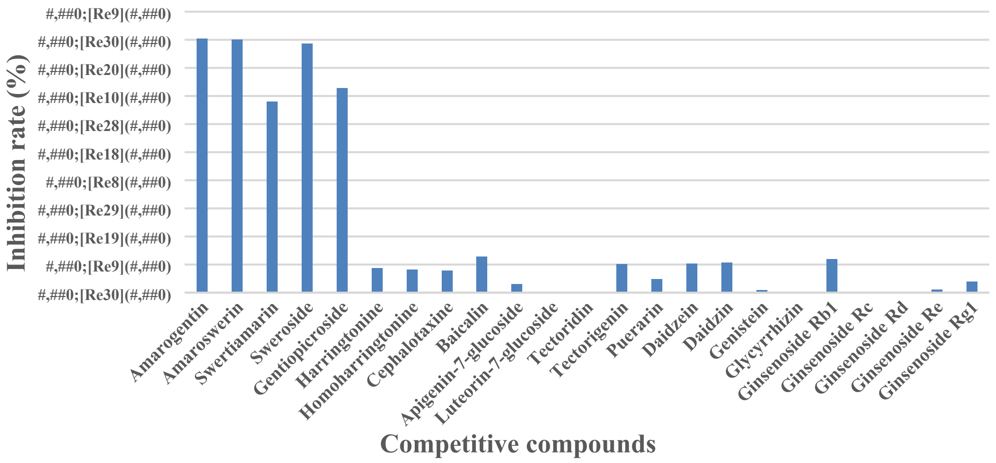

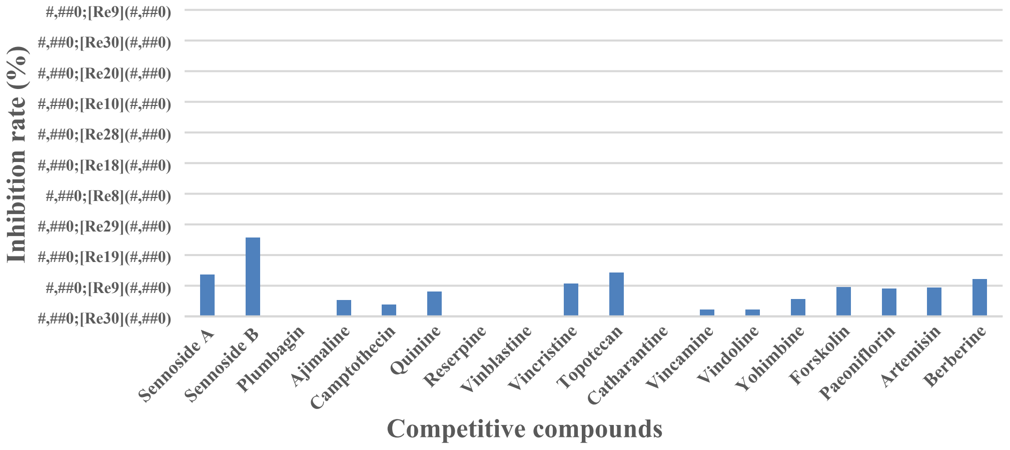

Fig. S2 Screening of test compounds for CRs test.

1. L.J. Xuan, H. Tanaka, S. Morimoto, Y. Shoyama, H. Akanuma and K. Muraoka, *Spectroscopy*, 2000, **14**, 85−92.
